# A Word on Introductory Lectures

**Published:** 1848-06

**Authors:** 


*A word on Introductory Lectures.*—At one time the annual orations delivered at our Universities, and places of education, were looked forward to with something like the eagerness which is felt when a new Prime Minister is about to announce the views of his government. They were regarded as expressions of progress—as exponents of advance, not only of the individual, but of the body to which he belonged, and as such gave the key-note to the session of study which was to follow. But now-a-days these introductories consist of the dullest platitudes, or the most egregious self-laudation. Students of medicine are addressed as though they had not the advantages of the education which a shop-keeper acquires at the Mechanics' Institute, and are gravely told of the importance of a knowledge of Anatomy, Chemistry, and Botany, to the study of Medicine; or, if these common-places are not dwelt upon, the orator endeavours to arrest the attention of his hearers by demonstrating to them how remarkably fortunate they have been in the choice of a school in which to study.—*Athenæum.*

